# Tuberculosis Trends — United States, 2014

**Published:** 2015-03-20

**Authors:** Colleen Scott, Hannah L. Kirking, Carla Jeffries, Sandy F. Price, Robert Pratt

**Affiliations:** 1 Epidemic Intelligence Service, CDC; 2 Division of Tuberculosis Elimination, National Center for HIV/AIDS, Viral Hepatitis, STD, and TB Prevention, CDC

In 2014, a total of 9,412 new tuberculosis (TB) cases were reported in the United States, with an incidence rate of 3.0* cases per 100,000 persons, a decrease of 2.2% from 2013 (1). Although overall numbers of TB cases and rates continue to decline, the percentage decrease in rate is the smallest decrease in over a decade (1). This report summarizes provisional TB surveillance data reported to CDC’s National Tuberculosis Surveillance System for 2014. TB cases and rates decreased among U.S.-born persons, and although the case rate also decreased among foreign-born persons,† there was an increase in total number of cases among foreign-born persons. The rate among foreign-born persons in the United States in 2014 was 13.4 times higher than among U.S.-born persons. Racial/ethnic minorities continue to be disproportionately affected by TB within the United States. Asians continue to be the racial/ethnic group with the largest number of TB cases. Compared with non-Hispanic whites, the TB rate among Asians was 28.5 times higher, whereas rates among non-Hispanic blacks and Hispanics were each eight times higher. Four states (California, Texas, New York, and Florida), representing approximately one third of the U.S. population, accounted for half of all TB cases reported in 2014. Continued progress toward TB elimination in the United States will require focused TB control efforts among populations and in geographic areas with disproportionate burdens of TB.

Health departments in the 50 states and the District of Columbia (DC) electronically report to CDC verified TB cases that meet the CDC and Council of State and Territorial Epidemiologists case definition for TB.§ Reports include the patient’s self-reported race, ethnicity (i.e., Hispanic or non-Hispanic), human immunodeficiency virus (HIV) status, information about diagnosis and treatment, and drug-susceptibility test results for *Mycobacterium tuberculosis* isolates. CDC calculates national and state TB rates overall and by racial/ethnic group using currently available U.S. Census Bureau population estimates (2). The Current Population Survey provides the population denominators used to calculate TB incidence rates and percentage changes according to national origin.¶ In 2014, 1.2% (117 of 9,412) of patients had unknown country of birth, and 0.5% (47 of 9,412) had unknown race/ethnicity. For this report, persons of Hispanic ethnicity might be of any race; non-Hispanic persons were categorized as black, Asian, white, American Indian/Alaska Native, Native Hawaiian or other Pacific Islander, or of multiple races.

The national TB incidence rate in 2014 was 3.0 cases per 100,000 persons, ranging by state from 0.3 in Vermont to 9.6 in Hawaii (median = 2.0) (Figure 1). Twenty-nine states and DC had lower rates in 2014 than in 2013; 21 states had higher rates. Ten states and DC had higher rates than the national average (Figure 1). In 2014, as in 2013, four states (California, Florida, New York, and Texas) reported >500 cases each. Combined, these four states accounted for 4,795 TB cases, or 50.9% of all U.S. cases in 2014.

Among U.S.-born persons, the number and rate of TB cases declined in 2014. The 3,114 TB cases in U.S.-born persons (representing 33.5% of all cases in persons with known national origin) indicated a 6.3% decrease in the number of cases compared with 2013 and a 64% decrease compared with 2000 (Figure 2). The TB rate of 1.1 per 100,000 U.S.-born persons represented a 6.8% decrease since 2013, and a 67.6% decrease since 2000.

In 2014, the disparity in TB incidence between U.S.-born and foreign-born persons continued to increase. The total number of TB cases among foreign-born persons in the United States increased with a total of 6,181 TB cases reported (66.5% of all cases in persons with known national origin), representing a 1.5 percentage point increase from 65% in 2013. Despite the increased number of cases among the foreign born in 2014, the TB rate of 15.3 per 100,000 among foreign-born persons decreased 1.5% from 2013 because of an increase in the immigrant population. The 2014 rate among foreign-born persons represented a 42.0% decrease from the rate in 2000. In 2014, 55.3% of foreign-born persons with TB originated from five countries: Mexico (1,268 TB cases [20.6%]), the Philippines (745 [12.1%]), Vietnam (498 [8.1%]), India (472 [7.7%]), and China (420 [6.8%]).

Although the incidence rate among Asians continues to be the highest among all racial/ethnic groups (28.5 times higher than the incidence rate among whites), the incidence rate among Asians decreased from 18.6 per 100,000 in 2013 to 17.9 in 2014 (Table). From 2013 to 2014, TB rates among Hispanics remained relatively constant whereas rates decreased among blacks, whites, and Asians. Conversely, TB rates among persons in the “other” racial category (including American Indian/Alaska Native, Native Hawaiian or other Pacific Islander, and multiple race) increased from 3.8 in 2013 to 4.3 in 2014. Among persons with TB, 96% of Asians, 76% of Hispanics, 42% of blacks, and 23% of whites were foreign born. Among U.S.-born persons, blacks (36.4% [1,134 of 3,114]) were the racial/ethnic group with the greatest number of TB cases and the largest disparity compared with U.S.-born whites.

HIV status was known for 86% of TB cases reported in 2014. Among persons with TB who had a known HIV test result, 6.3% (506 of 8,072) had a positive test result for HIV infection.

Among persons aged ≥15 years with TB, 99% had known homelessness status, long-term care status, and incarceration status. Among persons aged ≥15 years with TB, 5.5% of whom reported being homeless within the past year, 2.2% were residing in a long-term care facility at the time of TB diagnosis, and 4.2% were confined to a detention or correctional facility at the time of TB diagnosis.

Drug-susceptibility test results for isoniazid and rifampin were reported for 97.8% and 97.4% of culture-confirmed TB cases in 2012 and 2013, respectively, the most recent years for which complete drug susceptibility data are available. The percentage of the 7,367 TB cases that were multidrug-resistant (MDR) TB** in 2013 remained stable at 1.3% (96 cases), compared with 1.1% (86 of 7,620 cases) in 2012. The percentage of MDR TB cases among persons without a previous history of TB has remained stable at approximately 1.0% since 1997. In 2013, foreign-born persons accounted for 87 (90.6%) of the 96 MDR TB cases. One case of extensively drug-resistant (XDR) TB†† was reported in 2014 compared with four cases in 2013 and two cases in 2012, but some final susceptibility test results are pending for 2014.

## Discussion

Despite the continued decline in U.S. TB cases and rates since 1993, the 2.2% decrease from 2013 to 2014 to a rate of 3.0 per 100,000 still does not achieve the goal of TB elimination (1 case per 1,000,000) set in 1989 (3) and reaffirmed in 1999 (4). This decline in the rate of TB was the smallest decrease in more than a decade and suggests the need for ongoing evaluation of TB elimination strategies overall and within high-risk populations.

In 2014, the proportion of persons with TB who were foreign-born continued to increase. The higher proportion of TB cases occurring in foreign-born persons compared with U.S.-born persons illustrates the close relationship between the global TB burden and disease patterns in the United States. The established pattern of increasing proportions of TB cases occurring in the foreign-born population reaffirms the need to support and strengthen TB control efforts abroad, especially in the countries of origin of immigrants to the United States. This includes but is not limited to the countries contributing over half of the U.S. foreign-born patients in 2014 (i.e., China, India, Mexico, the Philippines, and Vietnam).

Additional efforts should also be made to prevent TB by finding and treating persons with latent *M. tuberculosis* infection (LTBI) among groups at high risk in the United States, including those who are foreign born (5). The majority of cases of TB disease that occur in foreign born patients result from reactivation of LTBI rather than newly acquired infection (6). Although the high number of foreign-born persons living in the United States precludes testing every one of them, priority should be placed on those at highest risk for reactivation, such as those who underwent screening for TB overseas and were determined to have LTBI before arrival in the United States and those born in sub-Saharan Africa or Southeast Asia (7). Additionally, patients who are immunocompromised should undergo LTBI screening and receive treatment when appropriate (5).

What is already known on this topic?

The incidence of tuberculosis (TB) within the United States has been declining since 1993. An increasing proportion of cases are among foreign-born persons.

What is added by this report?

Provisional data for 2014 show the number of active TB cases newly reported in the United States was 9,412, with an incidence of 3.0 cases per 100,000 persons. This is a 2.2% decrease from the rate in 2013. The rate among foreign-born persons was 13.4 times higher than that for U.S.-born persons.

What are the implications for public health practice?

Continued vigilance, surveillance, and active prevention measures are needed to reach the TB elimination goal of <1 case per 1 million persons. To continue making strides toward elimination, alignment of domestic TB control activities with international TB control initiatives is needed to address increasing disparities between U.S.-born and foreign-born persons. Treatment of persons at high risk with latent *Mycobacterium tuberculosis* infection is also needed to address this disparity.

Overall, 86% of TB cases in 2014 had known HIV status at TB diagnosis. All TB patients should have counseling and testing for HIV infection (8).

The findings in this report are subject to at least two limitations. First, reports of the number of TB cases and case rates for 2014 are provisional. Second, case rates are based on estimates of population denominators that might not all be recently updated. CDC’s annual TB surveillance report will provide final TB case numbers and rates for 2014 later in 2015.

Continued progress toward TB elimination in the United States will require ongoing surveillance and improved TB control in groups at high risk, especially racial/ethnic minorities. Alignment of domestic TB control activities with international TB control initiatives is needed to address increasing disparities in TB rates between U.S.-born and foreign-born persons. Focused treatment of LTBI also is needed to prevent TB in all groups at high risk.

## Figures and Tables

**FIGURE 1:**
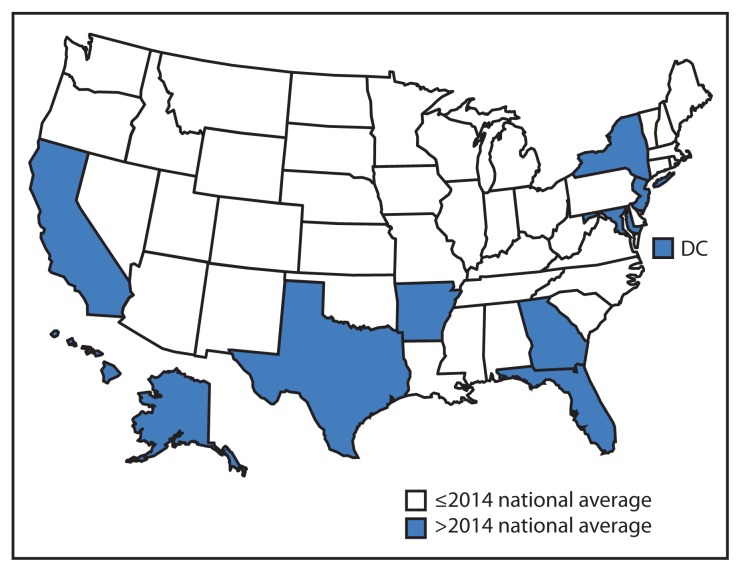
Incidence* of tuberculosis cases, by state and national average — United States, 2014^†^ * Per 100,000 population. ^†^ Data are provisional.

**FIGURE 2:**
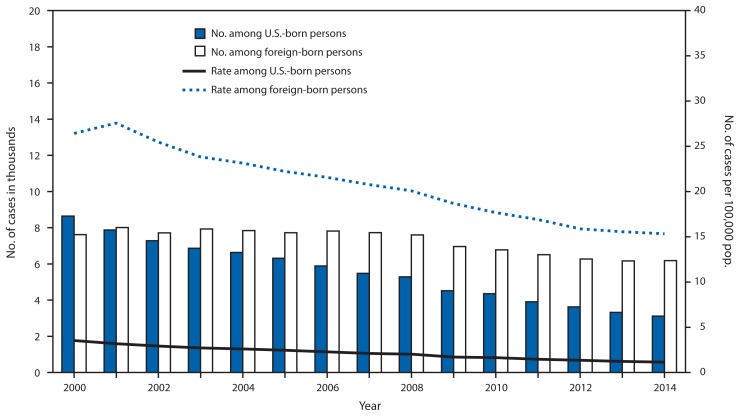
Number and rate* of newly diagnosed tuberculosis (TB) cases among U.S.-born and foreign-born persons, by year reported — United States, 2000–2014^†^ **Source:** National TB Surveillance System. * Per 100,000 population. ^†^ Data updated as of February 13, 2015. Data for 2014 are provisional.

**TABLE:** Number and rate* of tuberculosis cases and percentage change, by race/ethnicity — United States, 2013 and 2014†

	2013		2014		(%) change from 2013 to 2014	
			
Race/Ethnicity	No.	Rate	No.	Rate	No.	Rate
**Hispanic**	2,697	5.0	2,760	5.0	(2.3)	(0.5)
**Non-Hispanic**						
Black	2,089	5.3	1,996	5.1	(−4.5)	(−5.2)
Asian	2,989	18.6	2,961	17.9	(−0.9)	(−3.4)
White	1,424	0.7	1,247	0.6	(−12.4)	(−12.5)
Other§	341	3.8	401	4.3	(17.6)	(15.0)
Unknown	27	—	47	—	—	—
**Total**	**9,567**	**3.0**	**9,412**	**3.0**	**(**−**1.6)**	**(**−**2.2)**

* Per 100,000 population.

† Data for 2014 are provisional.

§ Includes persons reported as American Indian/Alaska Native (2014: 115 cases, rate = 4.9; 2013: 127 cases, rate = 5.5); Native Hawaiian or other Pacific Islander (2014: 94 cases, rate = 17.4; 2013: 60 cases, rate = 11.3); and of multiple races (2014: 192 cases, rate = 3.0; 2013: 154 cases, rate = 2.5).

## Footnotes

* In 2013, the overall U.S. TB rate was 3.02 per 100,000 persons; this declined to 2.95 per 100,000 persons in 2014.

† Includes persons born outside the United States in American Samoa, the Federated States of Micronesia, Guam, the Republic of the Marshall Islands, Midway Island, the Commonwealth of the Northern Mariana Islands, Puerto Rico, the Republic of Palau, the U.S. Virgin Islands, and U.S. minor and outlying Pacific islands.

§ Available at http://wwwn.cdc.gov/nndss/script/casedef.aspx?condyrid=876&datepub=1/1/2009%2012:00:00%20am.

¶ Additional information available at http://dataferrett.census.gov.

** Defined by the World Health Organization as a case of TB in a person with an *M. tuberculosis* isolate resistant to at least isoniazid and rifampin. Additional information available at http://whqlibdoc.who.int/publications/2010/9789241599191_eng.pdf.

†† Defined by the World Health Organization as a case of TB in a person with an *M. tuberculosis* isolate with resistance to at least isoniazid and rifampin among first-line anti-TB drugs, resistance to any fluoroquinolone (e.g. ciprofloxacin or ofloxacin), and resistant to at least one second-line injectable drug (i.e. amikacin, capreomycin, or kanamycin). Additional information available at http://whqlibdoc.who.int/publications/2010/9789241599191_eng.pdf.
